# Phosphate: An Anion Controlling Metabolic Functions

**DOI:** 10.3390/nu18152568

**Published:** 2026-08-06

**Authors:** Umberto Tarantino, Chiara Greggi, Beatrice Gasperini, Manuel Scimeca, Riccardo Iundusi, Elena Gasbarra, Maria Luisa Brandi

**Affiliations:** 1 Department of Clinical Sciences and Translational Medicine, University of Rome “Tor Vergata”, 00133 Rome, Italy; umberto.tarantino@uniroma2.it (U.T.); iundusi.riccardo@gmail.com (R.I.); elenagasbarra@tiscali.it (E.G.); 2 Department of Orthopaedics and Traumatology, “Policlinico Tor Vergata” Foundation, 00133 Rome, Italy; 3 Faculty of Medicine, University “Our Lady of Good Counsel”, 1005 Tirana, Albania; 4 Department of Sciences, University Roma Tre, Viale G. Marconi 446, 00146 Rome, Italy; beatrice.gasp95@gmail.com; 5 Department of Experimental Medicine, University of Rome “Tor Vergata”, 00133 Rome, Italy; manuel.scimeca@uniroma2.it; 6 FIRMO Foundation, 50139 Florence, Italy; marialuisa.brandi@unifi.it

**Keywords:** phosphate, mineral metabolism, phosphatonins, hypophosphatemia, hyperphosphatemia, biomineralization

## Abstract

Phosphate accounts for about 0.6% of body weight at birth and about 1% of body weight in adults. This anion is essential for the performance of many essential human cellular processes, including energy metabolism and cell signaling; it is also a key component of the phospholipid bilayer and nucleic acids structure. In addition, phosphate is responsible for maintaining acid-base balance within the cells, functioning of the nervous system and preserving bone integrity. Normal serum phosphate levels change throughout life, varying from the neonatal period, through adolescence, and stabilizing at adult values by the end of puberty. Serum phosphate homeostasis is maintained through dietary intake, intra- and intercellular shifts, and excretion/absorption/reabsorption processes mediated by Fibroblast Growth Factor 23, parathyroid hormone, and 1,25-(OH)_2_D_3_. Within this homeostatic network, dietary sources and current population-based intake data highlight a widespread oversupply in modern diets that can influence chronic disease outcomes. Ultimately, alterations in serum phosphate concentrations lead to numerous pathological conditions, both congenital and acquired. These conditions may involve either hypo- or hyperphosphatemia, each characterized by a wide spectrum of clinical manifestations, some of which primarily affect the musculoskeletal system. Starting from this evidence, the primary purpose of this review is to examine the phosphate-related pathological conditions from biological-molecular and clinical perspectives, while also considering targeted nutritional and clinical strategies for managing phosphate balance. Algorithms for management and differential diagnosis are also considered, thus providing a recent overview of a medical field that is becoming increasingly important for clinicians.

## 1. Introduction

Phosphate accounts for about 0.6% of body weight at birth and about 1% of body weight in the adult: 85% of phosphate is found in the skeleton and teeth, while the remaining 15% is found in extracellular fluid and soft tissues [1]. Phosphate is essential for numerous physiological processes, including cellular metabolism and signaling, and plays a crucial role for maintaining phospholipid bilayers and nucleic acids’ structure. Moreover, phosphate plays a key role in intracellular acid-base buffering, is essential for ATP-dependent cellular processes including those in neurons, and is necessary for the maintenance of bone integrity [2,3].

## 2. Materials and Methods

This narrative review was conducted through a structured literature search across the PubMed/MEDLINE, Scopus, and Web of Science databases, encompassing literature published up to January 2026. The search strategy utilized a combination of Medical Subject Headings (MeSH) and relevant text keywords joined by Boolean operators (AND, OR): “phosphate”, “mineral metabolism”, “phosphatonins”, “hypophosphatemia”, “hyperphosphatemia”, “biomineralization”, “FGF23”, “Klotho”, “vitamin D”, “parathyroid hormone”, “chronic kidney disease”, “tumor-induced osteomalacia”, and “X-linked hypophosphatemia”. The inclusion criteria comprised English-language, peer-reviewed articles, including original clinical and experimental studies, international guidelines, meta-analyses, and key narrative or systematic reviews. Articles were excluded if they were conference abstracts, letters to the editor, non-English publications, or focused on topics outside the scope of human phosphate metabolism.

## 3. Dietary Intake, Sources, and Recommendations

Dietary phosphorus is widely available in the human diet and exists in two distinct forms: organic and inorganic. Organic phosphate is naturally bound to proteins and is predominantly found in protein-rich foods such as dairy products, meat, fish, eggs, legumes, and nuts [4]. Its intestinal absorption is mediated by active transcellular transport via sodium-dependent phosphate co-transporter type IIb (NaPiIIb) and passive paracellular pathways, resulting in a moderate bioavailability of approximately 40% to 60% [5]. Notably, the absorption of organic phosphate from plant-based foods (e.g., cereals and legumes) is significantly lower (often below 30%) due to the presence of phytates, which human enzymes cannot efficiently degrade. In contrast, inorganic phosphate is extensively used as a food in modern industrialized food production, including ultra-processed foods, fast food, and carbonated beverages [6]. Unlike organic phosphate, inorganic phosphate salts are not bound to proteins; they dissociate rapidly in the intestinal lumen and are absorbed via non-saturable paracellular pathways with an exceptionally high bioavailability of nearly 90% to 100% [7]. Current clinical guidelines, such as those from the Dietary Reference Intakes (DRIs) and European authorities, recommend a daily allowance (RDA) of approximately 700 mg of phosphorus for healthy adults to maintain systemic mineral balance [8]. However, large-scale population-based studies (such as the National Health and Nutrition Examination Survey, NHANES) reveal that actual dietary intake in modern societies significantly exceeds these recommendations, frequently reaching 1200 to 1400 mg/day in Western populations. This substantial population-based dietary oversupply is driven primarily by the hidden consumption of inorganic phosphate additives. While observational studies suggest an association between high dietary phosphorus intake and adverse chronic outcomes, caution is required when inferring direct causality, as interventional evidence on hard clinical endpoints remains limited [9].

## 4. Phosphate Homeostasis

Serum phosphate levels are finely regulated and subjected to modulation by several hormones including parathyroid hormone (PTH), vitamin D hormonally active form, 1,25-dihydroxyvitamin D (1,25-(OH)_2_D_3_), and fibroblast growth factor 23 (FGF23). Phosphate is absorbed from the small intestine by NaPiIIb and filtered by the kidney where it is reabsorbed or excreted according to the body’s needs [10]. The main site of phosphate reabsorption is the proximal tubule of the kidney. In fact, about 70% of the filtered phosphate load is reabsorbed in the proximal convoluted tubule whereas approximately 10% is reabsorbed in the proximal straight tubule and distal segments of the nephron [1]. In the blood, phosphate concentration is maintained within a narrow range that varies with age and is about 0.9–1.5 mM in adults [11]. Extensive research has demonstrated that circulating humoral factors, collectively referred to as phosphatonins, can contribute to phosphate dysregulation [12,13]. Among these, FGF23 represents the dominant endocrine phosphaturic hormone, whereas other factors (including sFRP4, MEPE and FGF7) appear to exert more context-dependent or ancillary effects and are summarized in Table 1.

**Figure 1:**
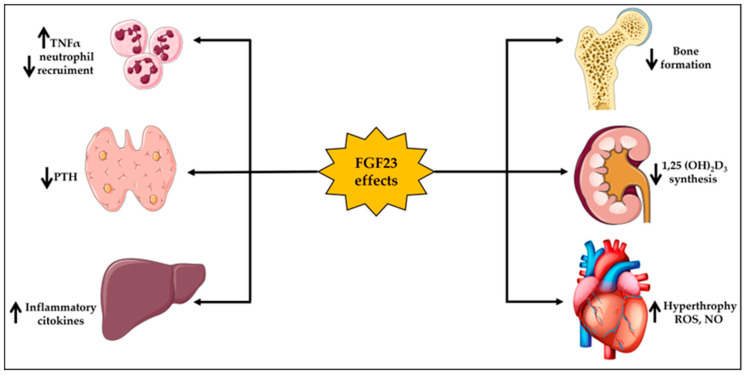
**Effects of FGF23 in humans.** FGF23 suppresses 1,25(OH)_2_D_3_, inhibits bone mineralization, induces cardiac hypertrophy, inhibits *PTH* expression and lowers PTH plasma levels; it stimulates tumor necrosis factor α (TNFα), and interferes with neutrophil recruitment. In the liver, FGF23 upregulates Interleukin-6 (IL-6) and *C*-reactive protein (CRP) expression.

These proteins may contribute to the global regulation of serum phosphate concentration directly by inhibiting renal phosphate transport or indirectly by controlling bone mineralization [12]. FGF23 is produced by osteocytes and osteoblasts: it suppresses 1,25(OH)_2_D_3_, inhibits bone mineralization, can induce cardiac hypertrophy and stimulates nitric oxide (NO) synthesis and reactive oxygen species (ROS) generation in human coronary endothelial cells [13,14,18]. In addition, FGF23 inhibits *PTH* expression and lowers PTH plasma levels through mitogen-activated protein kinase (MAPK) signaling. FGF23 has also been implicated in the modulation of innate immune responses, including effects on macrophage inflammatory signalling and neutrophil recruitment [19]. Under pathological conditions, the pro-inflammatory effects of FGF23 can be observed: in fact, in the liver, FGF23 upregulates Interleukin-6 (IL-6) and *C*-reactive protein (CRP) expression, promoting inflammation in chronic kidney disease (CKD) [18] (Figure 1).

FGF23 release is mediated by several factors such as elevated dietary phosphorus intake, phosphate serum levels, the activity of 1,25(OH)_2_D_3_, calcium concentration, erythropoietin, iron deficiency, metabolic acidosis, and the presence of inflammatory cytokines [14]. FGF23 exerts its biological activities through interaction with Fibroblast Growth Factor receptor (FGFR), of which 4 subtypes (FGFR1, FGFR2, FGFR3, FGFR4) have been identified [20]. FGFRs consist of an extracellular portion composed of three immunoglobulin-like domains (Ig1, Ig2, and Ig3), a transmembrane single-helix domain, and an intracellular domain with tyrosine-kinase activity. Between Ig1 and Ig2 lies an acidic amino acid sequence known as the “Acid box,” which does not directly bind FGF23 but may modulate ligand binding and receptor specificity. The actual binding of FGF23 occurs primarily through Ig 2 and Ig 3 domains, which constitute the ligand-recognition interface [21,22].

**Figure 2:**
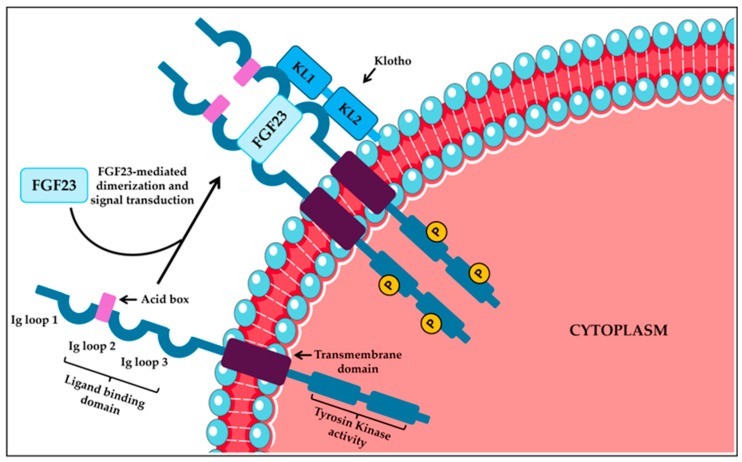
**FGF receptors.** FGFRs consist of an extracellular portion composed of three immunoglobulin-like domains, a transmembrane single-helix domain, and an intracellular domain with tyrosine-kinase activity. The three immunoglobulin-like domains (Ig loops 1, 2 and 3) have an acidic amino acid tract (the “Acid box”) between loop 1 and loop 2. This portion may participate in the regulation of FGF23 binding to FGFR. Ig loops 2 and 3 are the domains responsible for FGF23 binding. The endocrine activity of FGF23 is due to the interaction of the FGF23-FGFR complex with Klotho protein, whose function is performed by the extracellular portion composed of two domains KL1 and KL2.

According to literature data, FGF23 is able to interact with FGFR isoforms 1, 3 and 4. Specifically, at tissue level, the endocrine activity of FGF23 is mediated by the interaction of the FGF23-FGFR complex with Klotho protein. Klotho is highly expressed in the kidney and parathyroid gland, with its extracellular portion performing the critical cofactor function, which is composed of two domains, KL1 and KL2 [12] (Figure 2).

**Figure 3:**
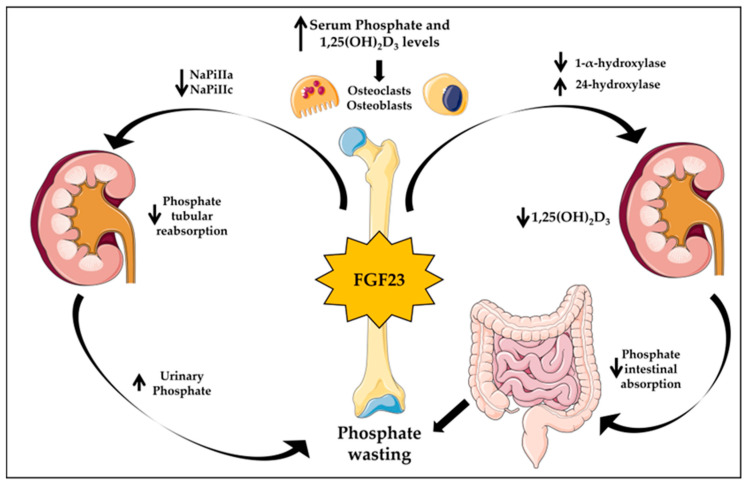
**Control of synthesis and metabolism of FGF23.** In response to elevated serum levels of phosphate and 1,25(OH)_2_D_3_, osteoblasts and osteoclasts release FGF23. The main site of action of FGF23 is the renal proximal tubule; at this level, FGF23 decreases the expression of Na-dependent phosphate transport protein a (NaPiIIa) and c (NaPiIIc), and increases urinary phosphate excretion, leading to phosphate wasting. At the same time, FGF23 exerts its control over phosphate homeostasis by reducing vitamin D active form levels, through inhibition of 1-α-hydroxylase and stimulation of 24-hydroxylase. Reduced renal biosynthesis of vitamin D active form therefore results in decreased intestinal reabsorption of phosphate.

The main site of action of FGF23 is the renal proximal tubule; at this level, FGF23 exerts its control over phosphate homeostasis by regulating the cellular expression of sodium-dependent phosphate transport protein a (NaPiIIa) and c (NaPiIIc), but also through the reduction of 1,25(OH)_2_D_3_ levels [23]. Regulation of the two NaPiII isoforms expression occurs through interaction with FGFR1, whereas inhibition of 1,25(OH)_2_D_3_ biosynthesis occurs via the interaction of FGF23 with FGFR isoforms 3 and 4 [24,25]. This process involves the functional inhibition of the enzyme 1-α-hydroxylase and the stimulation of 24-hydroxylase [25]. Reduced renal biosynthesis of the active form of vitamin D therefore results in decreased absorption of phosphate from the diet in the gut [26] (Figure 3).

MEPE encodes a glycosylated protein of approximately 60 kDa and appears to be a member of small integrin-binding ligand *N*-linked glycoproteins (SIBLINGs). MEPE knockout animals have showed increased bone density, suggesting that it may act as a mineralization inhibitor [12,14]. sFRP4 is a member of frizzled proteins family, containing a cysteine-rich domain with high homology to the extracellular domain of membrane-bound Wnt receptors [14]. It has been observed that sFRP4 inhibits sodium-dependent phosphate transport by decreasing NaPiIIa expression in the brush border membrane of the proximal tubule and on the surface of opossum kidney epithelial cells [15,16].

## 5. The Role of Phosphate in Life Stages

Phosphate concentrations are generally higher in infants and children than in adults, reflecting the increased requirements for skeletal growth and mineralization [27,28]. Age-dependent changes in intestinal and renal phosphate handling, including the expression of sodium–phosphate co-transporters, likely contribute to this physiological decline with maturation [27]. Phosphate also plays a primary role during the mineralization process that occurs during the growth spurt of the puberty stage [29]. In fact, there are numerous studies showing that phosphate levels differ between male and female sexes at different ages, suggesting a potential correlation between hormone levels and serum levels of phosphate [30]. Phosphate needs decrease from childhood, to puberty, to adulthood, although differences in serum levels are still observed between sexes, even after age 40. In fact, in the study by Koek and colleagues, sex-related differences in serum phosphate were found to be age-dependent: women older than 45 years showed higher serum phosphate levels than men. In addition, levels in women older than 45 years were higher than in women younger than 45 years [30]. In support of these findings, several studies have shown that postmenopausal women have higher serum phosphate levels than men of similar age [27].

Over the course of life, changes in serum phosphate levels are often accompanied by changes in serum calcium levels. Indeed, in addition to the functional decline of all body organs and the decline in energy metabolism that occur with aging, recently, a novel hypothesis has emerged that phosphate-calcium duet plays a primary role in directing metabolic senescence [28]. In line with this hypothesis, experimental models characterized by disruption of phosphate-regulating pathways, particularly the FGF23–Klotho axis, exhibit premature aging-like phenotypes that can be partially rescued by normalization of phosphate burden, supporting a contributory role of phosphate excess in organismal aging [31]. Phosphate plays an important role in energy metabolism regulation, both by regulating postprandial glucose accumulation in muscle and liver and the distribution of energy metabolites in response to brain and skeletal muscle demands [28]. The systemic distribution of phosphate depends exclusively on blood transport, which is strongly influenced by aging processes, due to structural changes that can occur in blood vessels. In elderly individuals therefore, the optimal reserve of inorganic phosphate is bone tissue. Bones are mechanically stimulated during every motor activity through the mechanoreceptor activity carried out by osteocytes [32]. However, in aging, bone tissue becomes less mechanosensitive, reducing its ability to respond to mechanical stimuli that normally regulate phosphate and calcium homeostasis. This impaired regulation contributes to decreased bone mineral density and an increased risk of osteoporosis [33]. Disruption of phosphate–calcium homeostasis also impairs the function of the nervous and muscular systems. Consequently, age-related alterations in phosphate–calcium metabolism can accelerate physiological aging and increase the risk of metabolic diseases associated with old age [28].

## 6. Conditions of Inherited and Acquired Hypophosphatemia

In adults, hypophosphatemia is defined as a serum phosphate concentration below the lower limit of the reference range (<2.5 mg/dL or <0.80 mmol/L). It is generally categorized by severity as follows: mild (2.0–2.4 mg/dL/0.65–0.79 mmol/L), moderate (1.0–1.9 mg/dL/0.32–0.64 mmol/L), and severe (<1.0 mg/dL/<0.32 mmol/L). In pediatric populations, hypophosphatemia must always be evaluated using age- and sex-adjusted reference intervals, as normal childhood baseline concentrations are significantly higher to support skeletal growth [31]. In this context, while hereditary forms dominate the genetic landscape, an inadequate dietary phosphorus intake or severe nutritional restriction is increasingly linked to detrimental chronic disease outcomes [34]. Although phosphorus is widely available in modern industrialized diets, prolonged insufficient dietary intake, often observed in conditions of severe malnutrition, chronic alcohol abuse, or prolonged eating disorders, triggers a state of chronic hypophosphatemia that significantly impacts long-term metabolic health [35]. Over time, a low dietary supply of phosphorus depletes intracellular adenosine triphosphate (ATP) pools, leading to a progressive decline in skeletal muscle function and chronic metabolic fatigue [35]. Furthermore, from a skeletal standpoint, sustained inadequate dietary phosphorus intake compromises the necessary mineral substratum for hydroxyapatite formation, leading to chronic bone remodeling imbalances. In the long term, this nutritional deficit manifests as osteomalacia in adults and metabolic bone diseases, which are characterized by severe bone pain, impaired physical mobility, a progressive reduction in bone mineral density, and a heightened risk of chronic skeletal fragility [36]. Human phosphate regulation occurs through FGF23–Klotho complex. Some experimental studies link excessive activity of FGF23–Klotho complex with increased phosphate excretion and consequent hypophosphatemia [37]. Diseases characterized by hypophosphatemia can be related or not to FGF23.

### 6.1. FGF23-Dependent Genetic Hypophosphatemic Rickets

#### 6.1.1. X-Linked Hypophosphatemia (XLH)

XLH (OMIM 307800) is the most common genetic form of rickets, with an incidence of approximately 1:20000, caused by dominant inactivating mutations in the phosphate-regulating gene with homologies to endopeptidases on the X chromosome (*PHEX*) gene. This gene is predominantly expressed in osteoblasts and teeth [38,39]. Pathogenic variants of *PHEX* result in increased circulating levels of FGF23, which cause a consequent decrease in NaPiIIa-c expression in proximal renal tubules, thereby impairing phosphate reabsorption from the glomerular ultrafiltrate. In addition, in regard to bone health, in patients with XLH, FGF23 suppresses 1α-hydroxylase and increases expression of 24-hydroxylase, interfering with vitamin D metabolism [40]. Therefore, these patients are characterized by dysregulation in bone metabolism, which manifests as an increased amount of osteoid and a prolonged delay in mineralization [41]. Thus, this condition is associated with abnormalities in skull shape (craniosynostosis, frontal bossing) and joints. Moreover, in children, weight-bearing bones are deformed due to rickets. As concern, the diagnosis is usually made in children who present with delayed gait, growth, and motor development, short stature, lower limb deformities, bone pain, and functional limitations in motor activities [42]. XLH in adults is mainly represented by mobility limitations, musculoskeletal pain, muscle weakness and hearing impairment [38]. Indeed, it is well known that phosphorus compounds play an important role in musculoskeletal function, especially energy-rich phosphates such as ATP, adenosine diphosphate (ADP), and phosphocreatine (PCr) [43]. Consequently, muscle weakness could be considered an intrinsic clinical feature of XLH, due to both skeletal deformities causing suboptimal ergonomics of the muscles and impaired muscle function due to deficient phosphate metabolism [44]. Burosumab, a human monoclonal antibody directed against FGF23, represents a targeted disease-modifying therapy for XLH that significantly improves renal phosphate reabsorption, serum phosphate levels, bone mineralization, and physical function compared to conventional therapy. However, its clinical application as a treatment of choice should be qualified according to patient age (approved in pediatric populations aged ≥1 year and in adults with active disease), specific clinical indications (e.g., progressive skeletal deformities, severe bone pain, or non-healing fractures), and regional reimbursement and availability constraints [45,46].

#### 6.1.2. Autosomal Dominant Hypophosphatemic Rickets (ADHR)

ADHR (OMIM 193100) represents a very rare form of inherited hypophosphatemic rickets, characterized by variable age of onset and significant clinical heterogeneity [47]. Affected individuals exhibit incomplete disease penetrance, with growth retardation, rickets, and lower limb deformities observed in patients with early-onset ADHR. In contrast, patients with adult-onset ADHR present with osteomalacia, weakness, bone pain, and fractures [48]. The onset of ADHR has been linked to four mutations in the subtilisin-like proprotein convertase (SPC) recognition site of the *FGF23* gene. These mutations render FGF23 resistant to enzymatic cleavage and leading to increased levels of intact, bioactive circulating FGF23. Iron deficiency increases the bioactivity of FGF23 by stimulating its gene transcription and reducing its degradation (due to ADHR mutation); penetrance is modulated by the state of iron depletion [49].

#### 6.1.3. Autosomal Recessive Hypophosphatemic Rickets (ARHR)

Homozygous mutation of Dentin Matrix Protein 1 (*DMP1*) has been identified as the cause of autosomal recessive hypophosphatemic rickets type 1 (ARHR 1) (OMIM 241520) [50]. DMP1 is a highly expressed non-collagenous acidic phosphoprotein found in teeth and bone, playing a crucial role in the processes of dentinogenesis and osteogenesis [51]. Furthermore, DMP1 is critical for the differentiation of osteoblast into osteocyte [52]. Because very few cases have been reported, the clinical features of this disorder have not yet been well defined, especially with regard to the bone microarchitecture [50]. Ni and colleagues studied five Chinese patients from three unrelated families with lower limb deformities and short stature [50].

Ectonucleotide pyrophosphatase/phosphodiesterase 1 (ENPP1) is considered a key regulator of skeletal and soft tissue mineralization [53]. It is known that biallelic loss-of-function mutations in *ENPP1* is associated with autosomal recessive hypophosphatemic rickets type 2 (ARHR2) (OMIM 613312) [54]. Subjects carrying *ENPP1* loss-of-function mutations have reduced blood levels of inorganic pyrophosphate (PPi), leading to calcium and phosphate precipitation and the formation of calcium-phosphate deposits, particularly in the internal elastic lamina of blood vessels, cartilage, and certain soft tissues [53]. Clinical manifestations of ARHR2 include skeletal deformities, short stature, lower limb deformities, early fusion of cranial sutures, bone fractures, bone and muscle pain, and, in some cases, progressive mixed hearing loss [55,56].

### 6.2. FGF23-Independent Genetic Hypophosphatemia

#### 6.2.1. Hereditary Hypophosphatemic Rickets with Hypercalciuria (HHRH)

HHRH (OMIM 241530) is a FGF23-independent form of hereditary hypophosphatemic rickets, caused by biallelic variants in the solute transporter family 34-member 3 (*SLC34A3*) gene. These mutations result in dysfunction of NaPiIIc in the proximal tubule, causing chronic renal phosphate loss [57]. In affected individuals, reduced tubular reabsorption of phosphate results in chronic hypophosphatemia, manifested by rickets or osteomalacia, limb deformity, short stature, bone pain, and muscle weakness [58]. Hypophosphatemia stimulates a compensatory increase in 1,25(OH)_2_D_3_, which increases intestinal calcium absorption, causing hypercalciuria. Excessive urinary calcium excretion promotes nephrocalcinosis, despite normal or low PTH levels [59].

#### 6.2.2. Vitamin D-Dependent Rickets

Vitamin D is converted in the skin after exposure to ultraviolet B rays in cholecalciferol (Vitamin D3) or ingested from the diet as vitamin D2 (ergocalciferol) or D3 [60]. Vitamin D requires two hydroxylations for activation: the first is catalyzed by Cytochrome P450 Family 2 Subfamily R Member 1 (CYP2R1), producing 25-hydroxyvitamin D (25-(OH)D); the second is catalyzed by 1-α-hydroxylase (Cytochrome P450 Family 27 Subfamily B Member 1, CYP27B1), resulting in the hormonally active form [61,62]. Defects in 25- or 1-α-hydroxylation lead to the inability to activate vitamin D, thus causing a state similar to vitamin D deficiency [63]. Vitamin D-dependent rickets (VDDR) is primarily a disorder of vitamin D activation or action that impairs intestinal calcium and phosphate absorption. Although secondary hyperparathyroidism triggered by hypocalcemia can induce transient urinary phosphate loss, VDDR is essentially a defect in intestinal absorption rather than a primary renal phosphate-wasting disorder [64,65]. VDDR type 1A (VDDR1A) (OMIM 264700) has been associated with loss-of-function mutations in *CYP27B1* gene [62]. VDDR1A is a disorder of vitamin D metabolism with autosomal recessive inheritance, typically presenting within the first year of life. VDDR1A manifests with neurological symptoms causing hypocalcemia, skeletal abnormalities, growth retardation, a low level of 1,25(OH)_2_D_3_, hypophosphatemia, increased ALP, secondary hyperparathyroidism and radiological abnormalities [64,66]. The absence of 1-α-hydroxylase reduces the levels of 1,25(OH)_2_D_3_, leading to impaired intestinal absorption of phosphate and calcium, which results in hypophosphatemia and hypocalcemia [65]. *CYP2R1* mutations cause vitamin D-dependent rickets type 1B (VDDR1B) (OMIM 600081) onset [62]. VDDR1B represents a severe form of rickets that occurs during childhood and is easily confused with classic vitamin D deficiency [67].

### 6.3. FGF23-Dependent Secondary Hypophosphatemia

Among the FGF23-dependent causes of secondary hypophosphatemia, tumor-induced osteomalacia (TIO) (OMIM 605380) stands out as a rare paraneoplastic syndrome and the most prevalent form of acquired hypophosphatemic osteomalacia [68]. It is a disease caused by small, often benign, slow-growing FGF23 expressing mesenchymal tumors [69]. Indeed, the biochemical features of TIO are represented by hypophosphatemia and increased or inappropriately normal levels of FGF23 [70]. Symptomatology includes muscular and skeletal manifestations, bone pain, weakness and fatigue, reduced mobility, and even multiple fractures, consequently leading to reduced mobility and the need for mobility aids [71]. Mesenchymal phosphaturic tumors represent, in most cases, the pathological entities underlying TIO. These tumors can be located anywhere in the body (lower limbs, upper limbs, head and neck, chest and abdomen, pelvis, spine, craniofacial bones, soft tissues, and more rarely in internal organs), involving both bone and connective tissues [72]. The latest non-surgical options for TIO treatment include burosumab, that improves hypophosphatemia and symptoms in patients with unresectable tumors, and, in selected cases, the use of cinacalcet or receptor analogues, to modulate phosphaturia [73].

### 6.4. FGF23-Independent Secondary Hypophosphatemia

Causes of FGF23-independent secondary hypophosphatemia include intracellular phosphate distribution due to insulin administration for the treatment of diabetic ketoacidosis, refeeding syndrome after a period of malnutrition, metabolic or respiratory alkalosis as a result of increased cytosolic phosphofructokinase activity, alcohol intake or withdrawal, glucose infusion, severe burns as a result of exudative leakage, hormonal agents, trauma, rapid cell proliferation, administration of catecholamines, beta-adrenergic agonists, and sodium bicarbonate [74,75,76,77]. Reduced phosphate intake attributable to severe nutritional restriction or due to severe malnutrition can also be a cause of FGF23-independent secondary hypophosphatemia [74]. Crohn’s disease, small bowel resection, bariatric surgery and celiac disease, on the other hand, represent causes of intestinal malabsorption of vitamin D, which in turn is responsible for a state of secondary hypophosphatemia [78]. Acquired Fanconi syndrome involves direct damage to the renal tubules caused by toxins or drugs, leading to generalized proximal tubulopathy and subsequent hypophosphatemia [75]. Primary and secondary hyperparathyroidism also represent causes of FGF-23-independent secondary hypophosphatemia. Specifically, vitamin D deficiency, secondary hyperparathyroidism due to chronic renal failure, and hypophosphatemia following kidney transplantation are examples of secondary hyperparathyroidism [79,80,81].

## 7. Conditions of Acute and Chronic Hyperphosphatemia

Hyperphosphatemia is defined as a clinical condition in which plasma phosphate levels exceed upper age-specific normal reference thresholds: >4.5 mg/dL (>1.45 mmol/L) in adults and >5.5 to 6.5 mg/dL (>1.8 to 2.1 mmol/L) in pediatric populations depending on age [82]. It can be caused by genetic or acquired diseases that disrupt the complex network regulating phosphate levels [83,84]. Intestine, bone, and kidneys are key organs involved in phosphate absorption, storage, and excretion. When phosphate intake exceeds normal levels due to exogenous sources, hormonal dysfunction, or impaired renal function, phosphate homeostasis is compromised, leading to hyperphosphatemia [85,86]. In this context, growing epidemiological and population-based data emphasize that the detrimental effects of hyperphosphatemia are not limited to advanced renal failure, but a high dietary phosphorus intake is progressively linked to adverse chronic disease outcomes even in individuals with normal renal function [87]. In population-based epidemiological cohorts, a sustained high dietary phosphorus intake has been observationally associated with increased cardiovascular risk and arterial stiffening. However, these findings represent epidemiological correlations rather than proven causal relationships, highlighting the need to distinguish between observational associations and verified clinical impacts. This clinical relationship is primarily mediated by the chronic overstimulation of the FGF23–Klotho endocrine axis and subtle, persistent elevations of serum phosphate within the high-normal range, which trigger endothelial dysfunction and low-grade systemic inflammation [88]. Furthermore, prolonged excessive phosphate intake disrupts systemic mineral homeostasis, inducing bone remodeling imbalances that contribute to reduced bone mineral density and a heightened risk of chronic skeletal diseases, including fragility fractures [89].

### 7.1. Acute Hyperphosphatemia Due to Transcellular Shift

#### 7.1.1. Tumor Lysis Syndrome (TLS)

In acute hyperphosphatemia, tumor lysis syndrome (TLS) is included, that can occur in patients undergoing cancer treatment, particularly prevalent in individuals with hematologic malignancies [90]. TLS is characterized by the rapid release of intracellular contents, such as nucleic acids, electrolytes, and other cellular components, into the bloodstream following tumor cell lysis. Cancer cells express a greater number of phosphate cotransporters and store more inorganic phosphate than normal cells, with implications for tumor growth, creating a detrimental cycle: elevated phosphate levels lead to acute renal insufficiency caused by calcium-phosphate precipitation in the renal tubules [91]. Furthermore, sustained hyperphosphatemia can result in calcium-phosphate deposition within connective tissues, significantly increasing the risk of metastatic calcification [92]. TLS can lead to metabolic imbalances and serious complications, including hyperphosphatemia, hyperkalemia, hyperuricemia, and hypocalcemia [93]. In fact, the massive release of intracellular phosphate from lysed tumor cells into the bloodstream can have several adverse effects on the body, with the resulting formation of calcium-phosphate complexes that can precipitate in the renal tubules, causing cardiac arrhythmias, cramps, muscle weakness, and tetany [94].

#### 7.1.2. Rhabdomyolysis

A severe medical condition characterized by the rapid breakdown of skeletal muscle tissue, which results in the release of phosphate from muscle cells [95,96]. The clinical signs of rhabdomyolysis include muscle pain, weakness, myoglobinuria, and elevated serum muscle enzymes [97]. This pathology can lead at potassium deficiency, hyperuricemia and hypercalcemia [98,99].

### 7.2. Acute Hyperphosphatemia Due to Increased Intestinal Phosphate Absorption

Through intestinal absorption following food ingestion, the body maintains adequate serum phosphate levels [100]. Once ingested and reaching the intestinal level, phosphate is absorbed through saturable sodium-dependent transcellular and a non-saturable sodium-independent paracellular pathway [101]. Due to the presence of NaPiIIb and sodium-dependent phosphate transporters (PiT-1) on the apical membrane of enterocytes, phosphate is internalized. It diffuses into the plasma circulation, with typical values ranging between 3 mg/dL to 4.5 mg/dL [102,103]. Intestinal phosphate co-transporters are hormonally regulated, with factors such as vitamin D, FGF23, and PTH modulating their expression and inhibiting phosphate absorption under conditions of phosphate excess. Phosphate is predominantly found in dairy products but is also present in fish, meat, and cereals, but phosphate bioavailability from legumes and cereals is comparatively low due to phytates [104]. However, phosphate intake in industrialized countries is increasing due to its presence in ultra-processed foods as phosphate additives [84]. A diet high in phosphate can lead to hyperphosphatemia with deleterious health consequences. Vitamin D excess, also known as vitamin D toxicity or hypervitaminosis D, can lead to several health problems, one of which is the hyperphosphatemia induced by intestinal phosphate absorption. However, dietary phosphorus intake does not usually cause acute hyperphosphatemia in individuals with normal renal function [105].

### 7.3. Acute Hyperphosphatemia Due to Reduced Renal Excretion

Acute kidney injury (AKI) leads to a rapid decline in glomerular filtration rate (GFR), which impairs phosphate excretion and can result in hyperphosphatemia, that can lead to a calcium-phosphate imbalance in the body [106]. Furthermore, in addition to calcium and phosphate dysregulation, alterations such as hyperparathyroidism, hyperuricemia, increased FGF23, and reduced expression of vitamin D and renal Klotho protein are observed in AKI and Chronic Kidney Disease (CDK). Specifically, secondary hyperparathyroidism during AKI depends on duration, severity, and degree of AKI, hyperphosphatemia and hypocalcemia [107,108]. According to KDIGO criteria, AKI is defined by an increase in serum creatinine ≥0.3 mg/dL within 48 h or ≥1.5 times baseline within 7 days, or by urine output <0.5 mL/kg/h for at least 6 h; however, several studies demonstrated that phosphate is an excellent candidate as a biomarker for the diagnosis and severity of the disease [109,110].

### 7.4. Acquired Chronic Hyperphosphatemia

Chronic Kidney Disease (CKD): CKD is the most common cause of chronic hyperphosphatemia [74]. In the early stages of CKD, serum phosphate levels are maintained within the normal range through increased production of FGF23, which acts to prevent hyperphosphatemia. The role of the FGF23 hormone is to reduce phosphate absorption in the intestine by inhibiting NaPiIIb expression and suppressing circulating vitamin D levels, thereby decreasing intestinal phosphate uptake [111]. In CKD, chronically high FGF23 levels exacerbate vitamin D deficiency, promoting hypocalcemia and secondary hyperparathyroidism [112]. When GFR decreases, these adaptive mechanisms are inadequate to maintain phosphate balance, and serum phosphate increases when GFR falls below 30 mL/min [113]. Additionally, FGF23, phosphate, and PTH levels rise significantly in the later stages of CKD, increasing the risk of cardiovascular events and mortality. Hyperphosphatemia is considered a determinant of the process of vascular calcification in patients with CKD [114]. Notably, population-based studies indicate that serum phosphate levels tend to increase with aging, even in individuals without overt CKD, suggesting that age-associated phosphate burden may contribute to vascular calcification and cardiovascular risk beyond the CKD setting [115].

In the presence of hyperphosphatemia, vascular smooth muscle cells (VSMCs) undergo a process of trans-differentiation towards an osteochondrogenic phenotype. This phenotypic remodeling promotes the establishment of a calcified microenvironment, which favors the nucleation and deposition of calcium-phosphate complexes [116,117]. The impaired renal function leads also to mineral and bone disorders (MBD) [118]. In CKD-MBD, altered bone and mineral metabolism and clinical events such as increased bone fragility, fractures, and related vascular and valve calcification are observed [115,119]. The process of vascular calcification in individuals with CKD is promoted not only by hyperphosphatemia but also by elevated FGF23 levels. As kidney function declines, serum phosphate levels rise, leading to increased secretion of FGF23 [112].

### 7.5. Acquired Hypoparathyroidism

Acquired hypoparathyroidism mainly arises from a decreased secretion of PTH, resulting in aberrant bone metabolism [120]. This condition in adults is predominantly attributed to surgical interventions involving the thyroid, parathyroid, adjacent neck structures, or neck dissection for malignancy. Therefore, acquired hypoparathyroidism usually stems from surgical damage. Additionally, clinical manifestations associated with acquired hypoparathyroidism included axillary hair loss, pubic hair loss, increased body hair growth, alopecia areata [121]. Low circulating PTH levels lead to the development of hyperphosphatemia, most likely due to increased renal reabsorption of phosphate. Hyperphosphatemia and hypocalcemia with an inappropriately low PTH level are characteristic features of hypoparathyroidism [122]. Calcium levels are tightly regulated in the body. When the parathyroid gland’s function fails, the production and release of PTH are impaired, resulting in an inadequate response to hypocalcemia. Consequently, there is insufficient mobilization of calcium from the skeleton, reduced renal resorption, and a failure to convert vitamin D to its active form, which is necessary for calcium absorption in the gut. PTH also inhibits the reabsorption of phosphate in the kidneys, and its absence can result in higher phosphate serum levels [123]. Hyperphosphatemia leads to the formation of ectopic calcifications in various regions of the body, particularly in the kidneys, brain, eyes, or blood vessels. This event is largely a consequence of treatment with calcium and 1,25(OH)_2_D_3_. Normal bone metabolism is altered, and bone mass increases. Additionally, Shoback and colleagues found that individuals with hypoparathyroidism experience a reduced quality of life and an increased risk of kidney stones, kidney calcifications, and kidney failure [124].

### 7.6. Genetic Hyperphosphatemic Disorder

#### 7.6.1. Genetic Hypoparathyroidism

Numerous genetic mutations can cause hypoparathyroidism, with hyperphosphatemia being a secondary issue associated with other significant clinical symptoms. However, only a small percentage of individuals are affected by genetic hypoparathyroidism, that affect the development or function of the parathyroid glands [125]. Genetic hypoparathyroidism can be caused by the 22q11.2 deletion syndrome, also known as DiGeorge syndrome (OMIM 188400). This syndrome is characterized by a broad clinical spectrum that includes congenital heart defects, craniofacial abnormalities, thymic hypoplasia with variable immunodeficiency, and neurodevelopmental disorders. Hypocalcemia occurs in approximately 50% of affected individuals and is primarily due to hypoparathyroidism resulting from abnormal development or hypoplasia of parathyroid glands [126,127]. Some genetic mutations can affect the embryological development of the parathyroid glands, leading to isolated hypoparathyroidism. The most common cause is a loss-of-function mutation in the glial cells missing transcription factor 2 (*GCM2*) gene, which plays a crucial role in the development of the parathyroid glands [128,129]. Autosomal dominant hypocalcemia falls under the category of genetic hypoparathyroidism [74]. Autosomal dominant hypocalcemia type 1 (ADH1) is a genetic disorder characterized by increased activity of the *CASR* gene product, which plays a key role in maintaining systemic calcium homeostasis. This results in hypocalcemia, low PTH levels, elevated urinary calcium, and hyperphosphatemia [130]. While ADH1 is often inherited, de novo mutations are also relatively common [128]. The clinical phenotype ranges from seizures in childhood to asymptomatic mild hypocalcemia in adulthood. Altered calcium-phosphate homeostasis determines severe complications, which may include renal dysfunction due to nephrocalcinosis or lithiasis, ectopic calcifications of the basal ganglia, posterior subcapsular cataracts, low bone turnover, and increased bone density [131].

#### 7.6.2. Hyperphosphatemic Familial Tumoral Calcinosis

Hyperphosphatemic familial tumoral calcinosis (HFTC) is an autosomal recessive disorder characterized by hyperphosphatemia and metastatic calcification, primarily affecting soft tissues around large joints. HFTC is caused by homozygous or compound heterozygous inactivating mutations in *FGF23*, UDP-*N*-acetyl-alpha-d-galactosamine, polypeptide *N*-acetylgalactosaminyltransferase 3 (*GALNT3*), or Klotho (*KL*) genes. Deficiency of intact FGF23 leads to increased renal phosphate reabsorption and elevated production of 1,25(OH)_2_D_3_, which in turn enhances intestinal absorption of calcium and phosphate. The resulting hyperphosphatemia promotes the formation of ectopic calcium-phosphate deposits in soft tissues. FGF23 knockout mouse models demonstrate that the absence of FGF23 action results in hyperphosphatemia and elevated serum levels of 1,25(OH)_2_D_3_. These mice also develop ectopic vascular and soft tissue calcifications, accompanied by a reduced lifespan [132].

## 8. Algorithm for the Management of Hypophosphatemia

**Figure 4:**
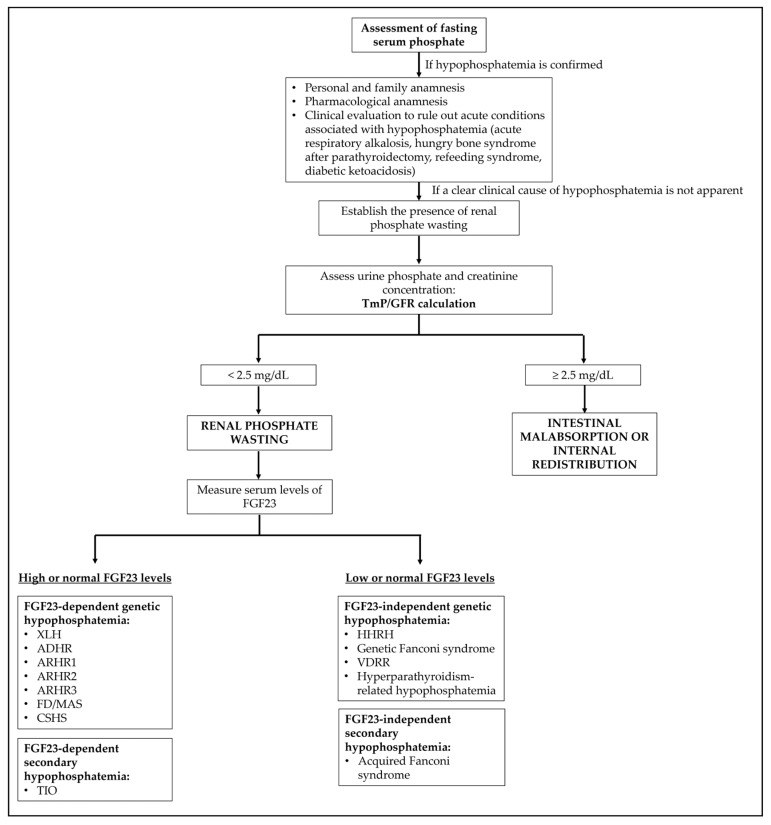
**Diagnostic and management algorithm for hypophosphatemia.** Differential diagnosis based on clinical history, tubular phosphate reabsorption capacity (TmP/GFR), and circulating FGF23 levels to distinguish between renal phosphate wasting, intestinal malabsorption, and intracellular redistribution. Diagnostic cut-offs and decision thresholds pertain to adult reference values. In pediatric patients, age- and sex-specific reference intervals for serum phosphate and TmP/GFR must strictly be applied. TmP/GFR: ratio tubular phosphate reabsorption rate (TmP) to glomerular filtration rate; FGF23: Fibroblast Growth Factor 23; XLH: X-linked hypophosphatemia; ADHR: Autosomal Dominant Hypophosphatemic Rickets; ARHR: Autosomal Recessive Hypophosphatemic Rickets; FD/MAS: Fibrous Dysplasia/McCune-Albright Syndrome; CSHS: Cutaneous-Skeletal Hypophosphatemia Syndrome; TIO: Tumor-Induced Osteomalacia; HHRH: Hypophosphatemic Rickets with Hypercalciuria; VDDR: Vitamin D-Dependent Rickets.

Clinical evaluation of hypophosphatemia should focus primarily on identifying the causes responsible for the decrease in serum phosphate levels, which is essential for the correct approach to the patient and for establishing the most appropriate therapeutic treatment [133,134]. Serum phosphate levels have a circadian pattern and vary substantially after meals, so serum phosphate should be assessed on an empty stomach, taking into account normal ranges that are gender- and age-specific [135]. If serum phosphate level assessment confirms hypophosphatemia, the next step should be to conduct a thorough family and personal history. It is necessary to check family history and diet before calculating tubular phosphate reabsorption rate (TmP) to glomerular filtration rate (TmP/GFR), in order to rule out transient causes of hypophosphatemia that do not require renal investigations. This should focus on determining the age of symptom onset, identifying any dental abnormalities, and investigating a history of nephrocalcinosis or related conditions [136]. In addition, this evaluation should be accompanied by a concurrent complete medication history to identify the use of any medications sometimes associated with hypophosphatemia (phosphate-binding antacids, antiretroviral drugs, intravenous iron, chemotherapeutic agents). Finally, a thorough clinical evaluation should be conducted to rule out any acute clinical conditions that could be responsible for the observed reduction in phosphate levels including acute respiratory alkalosis, hungry bone syndrome after parathyroidectomy, refeeding syndrome, diabetic ketoacidosis [36]. Only after excluding these potential contributors, if the clinical cause of hypophosphatemia is unclear, the next step is to verify a renal phosphate wasting, which can be done by calculating TmP/GFR [40,133]. This step is important to differentiate reduced intestinal phosphate absorption, internal redistribution or urinary loss [137]. To ensure an accurate assessment of TmP/GFR, serum and urine samples for phosphate and creatinine must be collected simultaneously (paired) under strict fasting conditions, as non-fasting or unpaired samples can lead to significant diagnostic misinterpretation due to circadian variations in phosphate handling. If urinary phosphate leakage is confirmed, the differential diagnosis is made on the basis of serum FGF23 levels [138]. In this context, circulating FGF23 levels should be evaluated using either intact (iFGF23) or *C*-terminal (cFGF23) immunoassays. While intact assays selectively detect the full-length biologically active peptide, *C*-terminal assays quantify both the intact hormone and its cleavage products; thus, results must always be interpreted according to assay- and laboratory-specific reference thresholds (Figure 4).

**Figure 5:**
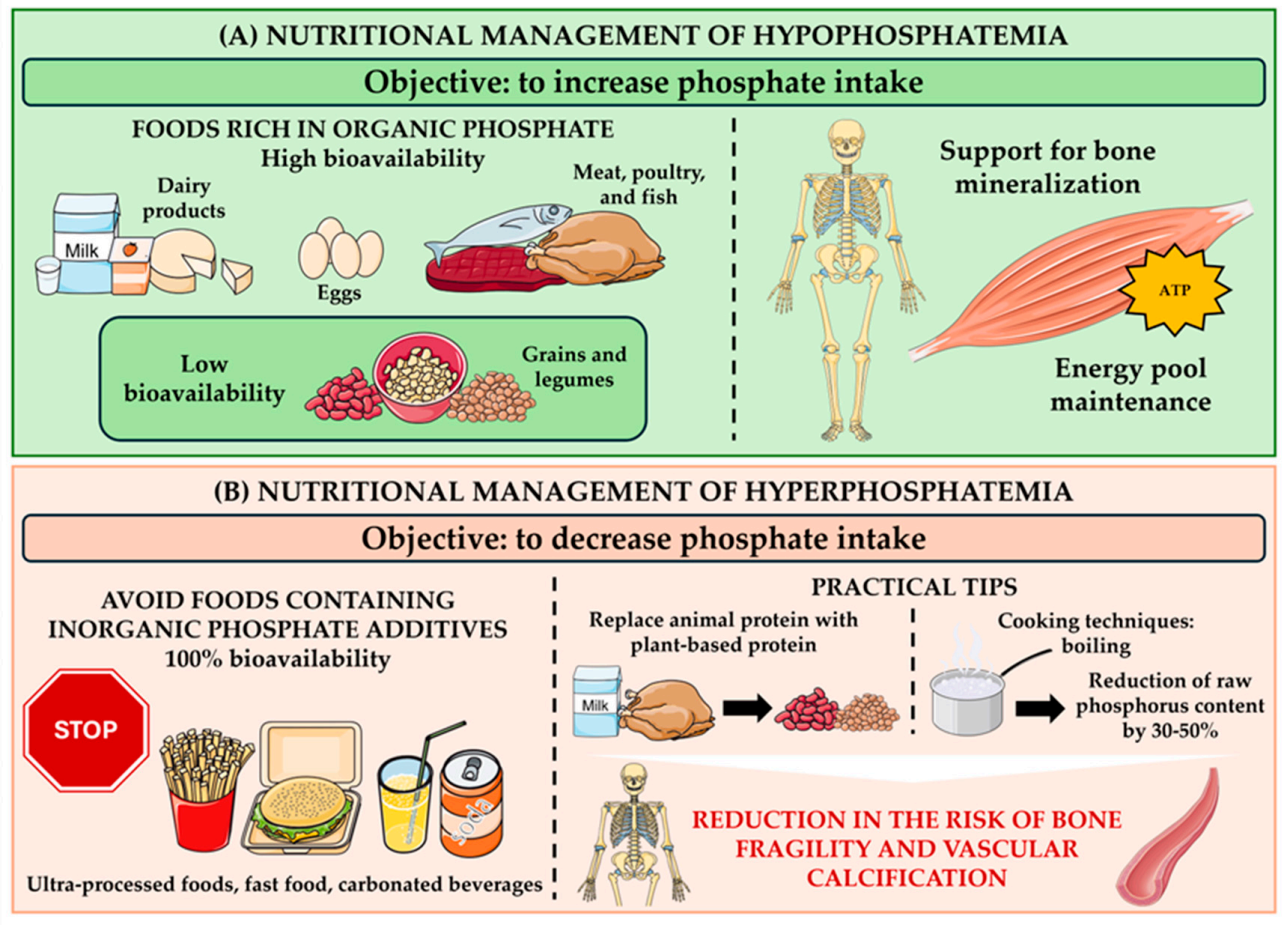
**Nutritional management of phosphate imbalances.** (**A**) Dietary approaches to elevate serum phosphate levels. Organic phosphate sources are classified based on their bioavailability, distinguishing high-bioavailability options (dairy, eggs, meat, poultry, and fish) from low-bioavailability plant sources (grains and legumes). The clinical relevance is tied to essential physiological functions, specifically supporting bone mineralization and maintaining the cellular ATP energy pool. (**B**) Dietary restrictions and practical interventions aimed at reducing phosphate burden. They include avoidance of ultra-processed items, fast food, and carbonated drinks due to the 100% bioavailability of their inorganic additives. To mitigate risks such as bone fragility and vascular calcification, it is suggested to replace animal proteins with plant-based alternatives and utilizing boiling techniques, which can effectively reduce raw phosphorus content by 30% to 50%.

In cases of mild-to-moderate hypophosphatemia, dietary replenishment represents the primary and safest approach before considering intravenous therapy, which carries risks of overcorrection and osmotic shifts [139]. Nutritional interventions focus on maximizing the intake of foods containing highly bioavailable organic phosphorus, particularly dairy products (milk, yogurt, cheese), eggs, meat, poultry, and fish, where phosphate is bound to proteins and readily absorbed via active and passive transport mechanisms [7]. In specific clinical scenarios such as refeeding syndrome, nutritional strategy must carefully balance phosphorus replacement with restricted carbohydrate intake; a rapid introduction of glucose can trigger massive insulin release, shifting extracellular phosphate into the intracellular compartment and drastically worsening hypophosphatemia [140]. Therefore, a gradual caloric titration (“start low and go slow”) combined with early dietary or oral phosphorus supplementation is essential to safely restore systemic phosphate balance [141]. Conversely, in cases of severe hypophosphatemia or in the presence of acute clinical manifestations such as neurological dysfunction, respiratory failure, or cardiac instability, dietary and oral approaches prove insufficient, and intravenous phosphate infusion becomes mandatory. This emergency therapy requires exceptionally rigorous monitoring of serum electrolytes, particularly potassium and calcium, to prevent fatal complications such as acute hypocalcemia, ectopical tissue calcification, and cardiac arrhythmias induced by rapid osmotic shifts. Once serum levels are stabilized and the critical phase of neuromuscular toxicity has been resolved, a gradual transition toward oral nutritional replenishment is initiated, reintroducing highly bioavailable foods to progressively restore depleted intracellular pools [139] (Figure 5).

## 9. Algorithm for the Management of Hyperphosphatemia

The management of hyperphosphatemia in patients requires a comprehensive and individualized approach, considering various factors such as underlying causes, severity, and patient-specific characteristics [142,143]. Hyperphosphatemia poses a significant challenge due to its association with heightened mortality and morbidity, including vascular calcification, cardiovascular complications, and secondary hyperparathyroidism [114]. Historically, guidelines from the Kidney Disease Outcomes Quality Initiative (KDOQI) recommended maintaining serum phosphate within a rigid target range of 3.5–5.5 mg/dL for CKD stages 3–5. However, updated Kidney Disease: Improving Global Outcomes (KDIGO) guidelines recommend a stepwise approach aimed at preventing or reducing progressive or sustained hyperphosphatemia toward normal laboratory reference levels in CKD stages G3a-G5D, rather than targeting a universal fixed range [144,145]. Moreover, age-dependent physiological thresholds must be considered: normal serum phosphate ranges from 2.5 to 4.5 mg/dL (0.80–1.45 mmol/L) in adults, whereas pediatric reference intervals are substantially higher to support skeletal growth, ranging from 4.5 to 6.5 mg/dL in infants and 4.0 to 5.4 mg/dL in children, before gradually declining to adult levels after puberty [11,27]. Once hyperphosphatemia has been confirmed, renal function must be assessed to support accurate diagnosis and treatment planning. In patients with impaired renal function, hyperphosphatemia is most commonly due to acute or chronic kidney failure, resulting in reduced renal phosphate excretion [82]. Conversely, in patients with apparently preserved renal function, urinary phosphate elimination should be evaluated to distinguish between renal phosphate retention, phosphate overload, and endocrine-mediated mechanisms [82]. This involves measuring urinary phosphate excretion to determine whether the kidneys are appropriately eliminating phosphate or if other mechanisms are involved [82]. To identify the factors contributing to hyperphosphatemia, it is necessary to assess PTH and 1,25(OH)_2_D_3_ levels [146,147]. High levels of PTH represent a physiological or compensatory response aimed at increasing renal excretion of phosphate, whereas reduced levels of circulating 1,25(OH)_2_D_3_ decrease intestinal phosphate absorption, thereby altering systemic phosphate balance [148,149]. Once the mechanisms have been determined, appropriate therapeutic strategies can be selected. Management relies on dietary phosphorus restriction and phosphate binders [150,151]. Nutritional strategies should prioritize the avoidance of ultra-processed foods, fast foods, and carbonated beverages containing inorganic phosphate additives, which are rapidly absorbed in the intestinal lumen with a bioavailability near 100% [4]. Conversely, patients should be encouraged to consume plant-based proteins (such as legumes and grains). Additionally, specific culinary interventions, such as boiling meat and fish in abundant water, can significantly reduce raw phosphorus content by 30–50% through leaching without affecting protein quality, thereby providing a practical method to mitigate phosphate burden while preserving nutritional status [152] (Figure 5).

Calcium-based binders are effective but may increase calcium load and vascular calcification risk, whereas non-calcium binders (e.g., sevelamer, lanthanum carbonate) provide comparable phosphate control without this limitation [153,154]. Given that CKD represents the most frequent underlying cause of persistent hyperphosphatemia in clinical practice, non-pharmacological and pharmacological interventions must be tailored to the specific CKD stage. In non-dialysis CKD stages 3a to 5, initial management prioritizes dietary phosphorus control alongside the maintenance of adequate protein intake to prevent protein-energy wasting [144]. When hyperphosphatemia persists despite dietary intervention, phosphate binder therapy is initiated. Calcium-based phosphate binders (such as calcium carbonate and calcium acetate) are effective and low-cost options; however, to minimize the risk of arterial calcification and hypercalcemia, international guidelines recommend restricting their dose and avoiding them altogether in the presence of vascular calcification or adynamic bone disease [144,155]. In these higher-risk scenarios, or in patients with advanced CKD (stages 4–5 and 5D on maintenance dialysis), non-calcium-based binders-including sevelamer carbonate, lanthanum carbonate, and iron-based formulations (sucroferric oxyhydroxide and ferric citrate) are preferred [145,156]. Iron-based binders offer the additional clinical advantage of improving iron parameters and reducing the need for intravenous iron and erythropoiesis-stimulating agents in dialysis populations [157]. Conversely, aluminum-containing binders are strictly restricted to short-term rescue use due to severe long-term bone and central nervous system toxicity [158].

**Figure 6:**
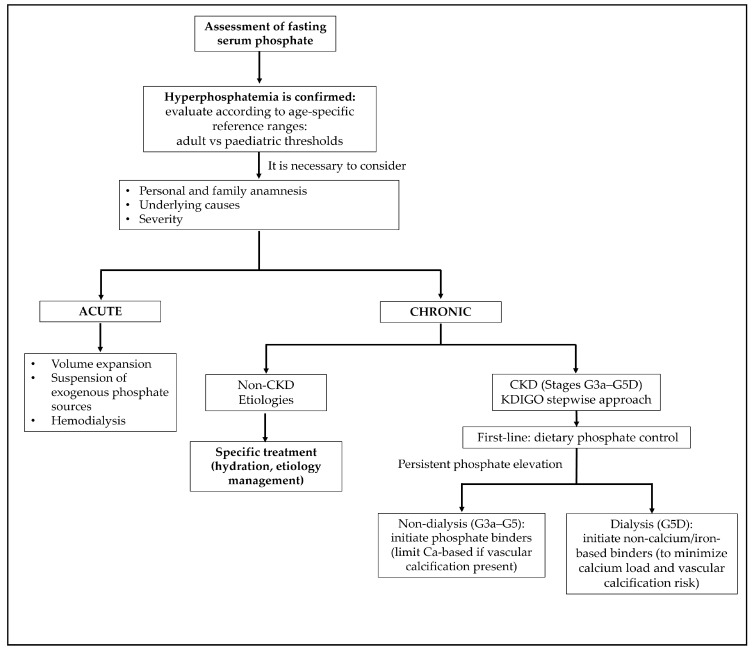
**Diagnostic and management decision tree for hyperphosphatemia.** After confirming the status of hyperphosphatemia, a distinguishing between acute or chronic hyperphosphatemia status, guidelines from the Kidney Disease Outcomes Quality Initiative (KDOQI) recommend specific treatment strategies for acute hyperphosphatemia and chronic hyperphosphatemia.

In selected settings, dialysis and therapies targeting CKD–MBD (vitamin D analogues and calcimimetics) may be required. Surgical interventions, such as excision of large calcium-phosphate deposits or parathyroidectomy for tertiary hyperparathyroidism, may be necessary in specific cases, highlighting the importance of multidisciplinary collaboration. In any case, surgical excision of calcium-phosphate deposits is rarely performed and should be framed accordingly. Regular monitoring, as advocated by KDIGO guidelines, aids in early detection of abnormalities, informing appropriate adjustments to treatment strategies and emphasizing personalized care in hyperphosphatemia management [145]. Treatment of hyperphosphatemia depends on factors such as its cause, onset rate, and renal function. Acute hyperphosphatemia warrants prompt intervention, including volume expansion and suspension of exogenous phosphate sources. Severe cases may necessitate hemodialysis. Chronic hyperphosphatemia management involves dietary restriction and phosphate binders, with potential initiation of combination therapy in cases of persistently elevated phosphate levels. Although phosphate binders effectively lower serum phosphate levels (improving biochemical surrogates), robust evidence from prospective randomized controlled trials demonstrating that pharmacological phosphate reduction translates into improved hard clinical outcomes—such as cardiovascular events or overall survival, remains limited [145,153]. While several observational studies and meta-analyses suggest potential survival benefits with non-calcium binders compared to calcium-based agents, these findings should be interpreted cautiously due to potential residual confounding [153,154]. In fact, unlike widely used calcium-containing drugs, non-calcium binders do not increase calcium load. They may even improve bone health and help reduce vascular calcification. Furthermore, several studies have reported decreased vascular calcification and improved CKD-MDB status [159]. Thus, the management of hyperphosphatemia in CKD and End-Stage Renal Disease (ESRD) necessitates a multifaceted approach, encompassing dietary modifications, phosphate binders, alternative therapies, and surgical interventions when warranted. Personalized care, interdisciplinary collaboration, and regular monitoring are crucial in optimizing patient outcomes and mitigating complications associated with hyperphosphatemia (Figure 6).

Dosing regimens, titration strategies, and major drug interactions for the main classes of phosphate binders are summarized in Table 2.

## 10. Current Controversies and Unresolved Issues

Despite significant advances in understanding phosphate homeostasis, several fundamental clinical controversies remain unresolved. In dialysis populations, the optimal serum phosphate target is still debated: while epidemiological data consistently associate hyperphosphatemia with cardiovascular mortality, rigid normalization risks protein-energy wasting, leading current guidelines to advocate for broader, trend-based targets rather than strict limits [145]. Moreover, robust evidence from placebo-controlled randomized clinical trials proving that pharmacological phosphate lowering directly improves hard clinical outcomes (such as cardiovascular events or overall survival) remains surprisingly limited. In non-dialysis CKD, the utility and safety of early phosphate binder initiation also remain contentious, with concerns regarding vascular calcification, particularly with calcium-based agents. Furthermore, the clinical integration of novel therapeutic classes, such as the gut paracellular phosphate absorption inhibitor tenapanor, presents new questions regarding combination strategies and long-term compliance [160]. Finally, in rare FGF23-mediated disorders like TIO, while conventional oral phosphate and active vitamin D supplementation remain standard when resection fails, the anti-FGF23 monoclonal antibody burosumab offers a novel targeted approach, though its long-term cost-effectiveness and positioning relative to conventional therapy are still being established [161].

## 11. Conclusions

Phosphate is essential for cellular metabolism, signalling and bone mineralization. Systemic phosphate homeostasis is regulated primarily by PTH, 1,25(OH)_2_D_3_ and FGF23. Physiological requirements vary across the lifespan, with higher demands during growth and developmental stages. Deviations from normal phosphate levels underpin clinically relevant hypo- and hyperphosphatemic disorders, which may be inherited or acquired and FGF23-dependent or -independent. Noteworthy, emerging evidence suggests that phosphate should not be regarded solely as a mineral component of skeletal biology, but also as a potential pleiotropic modulator of aging-related vascular and metabolic processes. Within this framework, modern dietary patterns—characterized by a widespread oversupply of highly bioavailable inorganic additives from ultra-processed foods—frequently challenge systemic regulation and exacerbate chronic mineral imbalances. Lastly, clinical algorithms should focus on confirming the biochemical abnormality and applying targeted laboratory testing to define the underlying mechanism and guide management. In this comprehensive therapeutic landscape, personalized nutritional strategies, including the optimization of organic protein sources and specific culinary interventions like food boiling, serve as a fundamental, non-pharmacological support to safely restore systemic phosphate balance, minimize treatment complications, and preserve patient nutritional status.

## 12. Limitations of the Review

This narrative review has some inherent limitations that should be acknowledged. First, the clinical guidelines, dietary intake data, and population-based evidence cited herein rely primarily on studies conducted in developed Western countries, which may limit the generalizability of these findings to populations with different dietary habits or healthcare infrastructure. Second, this work focuses mainly on the biological, molecular, and pathophysiological mechanisms of phosphate disorders, omitting detailed analyses of the economic burden, cost-effectiveness of pharmacological treatments, and healthcare resource utilization. Finally, given the narrative nature of this overview, certain specific clinical topics or localized disease management strategies did not systematically include randomized controlled trials (RCTs), particularly in rare or emerging therapeutic areas where high-level evidence remains limited.

## Figures and Tables

**Table 1:** Phosphatonin hormones.

Phosphatonin	Expression	Effect	Model	References
**FGF23**	bone tissue	Decreases renal phosphate reabsorption by decreasing the expression of the co-transporters NPTIIa and NPTIIc on proximal renal tubules, through activation of its receptor FGFR1c; decreases 1,25(OH)_2_D_3_ production in the kidney by inhibiting the expression of 1-α-hydroxylase and by stimulation of 24-hydroxylase	Human	[14]
**MEPE**	bone tissue and teeth	Mineralization inhibition	Mice	[12,14]
**sFRP4**	endometrium and ovary	Inhibition of Na-dependent phosphate transport by decreasing NaPiIIa expression in the proximal tubule	Opossum Rat	[15,16]
**FGF7**	several tissues	Inhibition of Na-dependent phosphate transport in kidney epithelial cells and increased phosphaturia	Opossum Rat	[17]

FGF23: Fibroblast Growth Factor 23; MEPE: matrix phosphoglycoprotein; sFRP4: secreted frizzled-related protein 4; FGF7: Fibroblast Growth Factor 7; NPTIIa: sodium-dependent phosphate co-transporter type IIa; NPTIIc: sodium-dependent phosphate co-transporter type IIc; FGFR1c: Fibroblast Growth Factor Receptor 1c; 1,25(OH)_2_D_3_: 1,25-dihydroxy vitamin D3: NaPiIIa: Na-dependent phosphate transport protein a.

**Table 2:** Overview of dosing, titration regimens and drug interactions for phosphate binders.

Binder Class/Agent	Initial Starting Dose	Titration Regimen	Major Drug Interactions	References
**Calcium-Based** *Calcium Carbonate Calcium Acetate*	500–1000 mg (elemental Ca) per day with meals (or 667–1334 mg calcium acetate t.i.d. with meals)	Adjust every 2–4 weeks by 1 tablet/meal based on serum phosphate and calcium levels. Limit elemental Ca to ≤1500 mg/day	Decreases absorption of fluoroquinolones, tetracyclines, and thyroxine (separate by ≥2 h); Glucocorticoids reduce intestinal Ca absorption; concurrent use may impair overall efficacy	[144,145,151]
**Non-Calcium Polymeric** *Sevelamer* (*Carbonate*/*HCl*)	800–1600 mg t.i.d. with meals (depending on baseline serum phosphate level)	Increase by 1 tablet (800 mg) per meal every 2–4 weeks to achieve target phosphate levels	Binds ciprofloxacin, mycophenolate mofetil, and levothyroxine (separate administration by 2–4 h); PPIs may reduce sevelamer carbonate binding efficiency due to altered gastric pH	[151,154,159]
**Metal-Based** *Lanthanum Carbonate*	750–1500 mg/day in divided doses with meals (e.g., 250–500 mg t.i.d., chewable)	Titrate by 750 mg/day every 2–3 weeks until phosphate goals are met (max dose: 3750 mg/day)	Reduces bioavailability of tetracyclines, quinolones, and levothyroxine (separate by ≥2 h); Minimal interaction with PPIs, though gastric pH alterations should be monitored	[145,151,156]
**Iron-Based** *Sucroferric Oxyhydroxide* *Ferric Citrate*	*Sucroferric*: 500 mg (1 tab) t.i.d. with meals *Ferric Citrate*: 2 tablets (210 mg ferric iron/tab) t.i.d. with meals	Adjust by 1–2 tablets per day at 1–2 week intervals as needed to achieve control	Binds doxycycline, oral iron supplements, and alendronate (separate by 1–2 h); Concurrent glucocorticoid use may alter systemic iron absorption kinetics	[151,153,154,157]

**Abbreviations:** Ca, calcium; HCl, hydrochloride; mg, milligram; PPIs, proton pump inhibitors; t.i.d. (ter in die), three times daily.

## Data Availability

No new data were created or analyzed in this study. Data sharing is not applicable to this article.
